# Prehypertension in young physically active adults: early vascular alterations and implications for exercise-intensity prescription

**DOI:** 10.3389/fcvm.2026.1853384

**Published:** 2026-06-29

**Authors:** Gašper Turnšek, Simon Iskra, Armin Huso Paravlić

**Affiliations:** 1 Institute of Kinesiology, Faculty of Sport, University of Ljubljana, Ljubljana, Slovenia; 2 Institute for Kinesiology Research, Science and Research Centre Koper, Koper, Slovenia; 3 Faculty of Sports Studies, Incubator of Kinanthropology Research, Masaryk University, Brno, Czechia

**Keywords:** arterial stiffness, exercise-intensity prescription, prehypertension, vascular function, ventilatory thresholds

## Abstract

**Background:**

Prehypertension represents an early stage of vascular dysfunction and an important precursor of cardiovascular disease. It remains unclear whether early vascular alterations are already present in young, physically active adults and whether these alterations are accompanied by changes in aerobic capacity or inaccuracies in commonly used exercise-intensity prescription methods.

**Methods:**

Participants included 51 recreationally active young adults (age, 24.5 ± 2.6 years). They were categorized as normotensive (*n* = 26) or prehypertensive (*n* = 25). Central and peripheral hemodynamics were assessed using oscillometric pulse wave analysis and pulse wave velocity. Participants performed a maximal cardiopulmonary exercise test to determine maximal oxygen uptake ($\dot{V}O_{2\max}$) and ventilatory thresholds. Fixed relative intensity anchors based on percentage of maximal heart rate, heart rate reserve, and maximal oxygen uptake were evaluated for their ability to estimate exercise-intensity domains corresponding to ventilatory thresholds.

**Results:**

Prehypertensive participants exhibited higher aortic pulse pressure and carotid–femoral pulse wave velocity (both *p* < 0.001), indicating less favourable central vascular characteristics. In contrast, aerobic $\dot{V}O_{2\max}$, ventilatory thresholds, and exercise heart-rate responses were comparable between groups (all *p* > 0.05). Fixed relative intensity anchors showed limited accuracy in approximating exercise-intensity domains defined by ventilatory thresholds, with pronounced misclassification at the first ventilatory threshold (VT1) and better agreement at the second ventilatory threshold (VT2). Among the evaluated approaches, population-derived %$\dot{V}O_{2\max}$ anchors showed the lowest estimation error at VT1, whereas %HRmax anchors demonstrated the best agreement at VT2.

**Conclusion:**

In young, physically active adults, prehypertension is associated with early central vascular alterations despite preserved aerobic capacity. Commonly used fixed relative intensity anchors do not accurately reflect physiological exercise-intensity domains, highlighting the need for more individualized approaches to exercise prescription in this population.

## 1 Introduction

Prehypertension, defined as a blood pressure value ranking from 120 to 139/80–89 mmHg, is a highly prevalent preclinical condition that is increasingly recognized as an early indicator of vascular dysfunction and is associated with increased risk of developing hypertension (1–4). Even within this subclinical range, prehypertension is associated with adverse cardiovascular (CV) remodelling, early end-organ involvement, and elevated long-term CV mortality risk, underscoring its pathophysiological relevance and the importance of early intervention (5). Despite growing scientific attention, clinical practice remains predominantly oriented toward the management of hypertension, thereby creating a substantial preventive gap in individuals who have not yet crossed the diagnostic threshold but already exhibit heightened CV risk (6). In this context, lifestyle modification, particularly structured aerobic exercise, constitutes the primary non-pharmacological approach for mitigating risk trajectories associated with elevated blood pressure (7).

Aerobic capacity is a key determinant of CV health, contributing to enhanced hemodynamic regulation, reduced all-cause and CV mortality, and a lower likelihood of progression to hypertension (8). While extensive research has focused on how exercise capacity modulates blood pressure (9), considerably less attention has been directed toward the inverse relationship, whether elevated blood pressure within the prehypertensive range may impair aerobic capacity or alter physiological responses to exercise (10). Previous work reported no significant differences in maximal oxygen uptake ($\dot{V}O_{2\max}$) between normotensive and prehypertensive adults. However, submaximal indices of exercise performance, such as ventilatory thresholds, which may provide greater discriminatory sensitivity between these groups, have rarely been assessed (11). Furthermore, the vascular consequences of prehypertension may manifest differently in younger adults compared to older populations (12). Given the rising prevalence of prehypertension in otherwise healthy individuals, elucidating these cardiopulmonary differences is essential for informing targeted and preventive exercise interventions.

The basic tenets of exercise prescription are grounded in four fundamental principles: frequency, intensity, time, and type, collectively referred to as the FITT concept. Despite this well-established framework, accurately determining exercise intensity remains one of the primary methodological challenges in applied practice (13). Although intensity is the key driver of acute physiological stress and long-term adaptation, it is also the least standardized component of training (14). Unlike training volume, training intensity is difficult to classify due to its complexity and multiple factors it depends on, as well as the variety of methods used for its assessment, including physiological parameters (e.g., heart rate, $\dot{V}O_{2}$, lactate), and external load metrics (i.e., speed, power) (15). This challenge becomes even more pronounced in clinical populations, where interindividual heterogeneity and safety considerations further complicate intensity determination (16). Cardiopulmonary exercise testing (CPET) is widely regarded as the reference method for individualizing exercise-intensity prescription, with ventilatory thresholds derived from CPET often considered meaningful markers for defining intensity domains because they correspond to distinct transitions in metabolic regulation (11, 17, 18). Threshold-based prescription has been shown to provide superior precision and more homogeneous training adaptations compared with fixed relative intensity anchors such as the percentage of maximal heart rate (%HRmax), the percentage of heart rate reserve (%HRR), or the percentage of maximal oxygen uptake (%$\dot{V}O_{2\max}$) (13, 19, 20). However, identifying thresholds requires laboratory testing, which substantially limits feasibility and accessibility in many real-world settings. As a result, practitioners often rely on fixed relative intensity anchors (13, 21). Previous studies conducted in healthy and recreationally active adults have demonstrated that ventilatory thresholds occur across broad relative-intensity ranges when expressed as %HRmax, %HRR, or %$\dot{V}O_{2\max}$. Specifically, the first ventilatory threshold (VT1) has commonly been reported between 45%–74% $\dot{V}O_{2\max}$ and 60%–90% HRmax, whereas second ventilatory threshold (VT2) has typically been observed between 69%–96% $\dot{V}O_{2\max}$ and 75%–97% HRmax (11, 14, 19, 20, 22). Similarly, studies using %HRR-derived exercise intensity domains have reported substantial interindividual variability and frequent misclassification of ventilatory threshold boundaries (23). These findings further suggest that fixed relative-intensity anchors may inadequately reflect physiological exercise-intensity domains, even in apparently healthy adults (21). Importantly, the relative positioning of VT1 and VT2 appears to be influenced by factors such as cardiopulmonary fitness (24), biological sex, and CV health status (19, 22). This interindividual variability raises concern that fixed relative intensity anchors may misclassify true intensity, thereby attenuating the effectiveness of individualized training interventions (13, 19, 20). Although CPET remains the gold standard for identifying the VT1 and VT2, its widespread implementation is constrained, and discordance between guideline-based and threshold-based intensity classifications is increasingly recognized (11).

To date, no studies have systematically and directly compared cardiopulmonary parameters between normotensive and prehypertensive young adults, despite the growing clinical importance of early vascular alterations in this population. Therefore, the primary aim of this study was to examine differences in ventilatory thresholds, $\dot{V}O_{2\max}$, and markers of vascular health between normotensive and prehypertensive young adults. Additionally, to our knowledge, no research has evaluated the accuracy of commonly used fixed relative intensity anchors (%HRmax, %HRR, %$\dot{V}O_{2\max}$) for estimating VT1 and VT2 in this population. Moreover, their correspondence with existing guideline-based intensity classifications has not been systematically examined. Accordingly, the secondary aim was to evaluate the validity of these fixed relative intensity anchors in predicting ventilatory thresholds and to assess their agreement with current exercise-prescription guidelines while accounting for blood pressure status.

## 2 Materials and methods

### 2.1 Study population

The current dataset consists of pooled test results from two studies (NCT06669715 and NCT07048509), both conducted at the Exercise Physiology Laboratory, Institute of Sport, Faculty of Sport, University of Ljubljana. In total, 171 asymptomatic individuals underwent initial screening as part of the study protocols. All assessments were performed at the Faculty of Sport, University of Ljubljana between October 2024 and November 2025. The exclusion criteria were: (1) age <20 and >30 years; (2) presence of hypertension (systolic BP ≥ 140 mmHg, diastolic BP ≥ 90 mmHg, or current use of antihypertensive medication); (3) presence of any non-communicable chronic disease; and (4) lower-limb injury within the preceding six months. Following application of these criteria, 51 participants were eligible for inclusion. The sample consisted of apparently healthy females (*n* = 19) and males (*n* = 32). According to the McKay classification framework (25), participants were categorized as recreationally active or trained (Tier 1–2). Physical activity levels were assessed using the Global Physical Activity Questionnaire (GPAQ). All participants met current physical activity recommendations for health maintenance proposed by the World Health Organization (WHO) and the American College of Sports Medicine (ACSM), as they accumulated more than 1000 MET-minutes of physical activity per week (26, 27). Participant characteristics are presented in Table 1.

**Table 1:** Descriptive characteristics of the participants.

Characteristics	All (*N* = 51)	Normotension (*N* = 26)	Prehypertension (*N* = 25)	*p*—value	ES	95% CI	
						Lower	Upper
Demographics							
Age (years)	24.49 ± 2.64	24.38 ± 2.44	24.61 ± 2.89	0.758	−0.09	−0.63	0.46
Female, *n* (%)	19 (37.3)	15 (57.7)	4 (16)				
Male, *n* (%)	32 (62.7)	11 (42.3)	21 (84)				
GPAQ (METs/week)	6,669 ± 2,366	6,942 ± 2,501	6,406 ± 2,248	0.434	0.22	−0.33	0.77
Anthropometrics							
Height (cm)	174.37 ± 9.53	171.38 ± 9.37	177.47 ± 8.83	0.021*	−0.66	−1.21	−0.99
Weight (kg)	70.47 ± 11.13	66.94 ± 10.38	74.14 ± 10.87	0.019*	−0.67	−1.22	−0.11
BMI (kg/m^2^)	23.08 ± 2.50	22.70 ± 2.28	23.47 ± 2.70	0.272	−0.31	−0.85	0.24
Clinical measures							
Systolic blood pressure (mmHg)	119.69 ± 11.18	111.21 ± 6.26	128.53 ± 8.01	<0.001*	−2.36	−3.07	−1.64
Diastolic blood pressure (mmHg)	72.04 ± 6.33	71.17 ± 5.07	72.94 ± 7.54	0.329	−0.27	−0.81	0.27
Mean arterial pressure (mmHg)	82.4 ± 6.74	79.6 ± 5.93	85.4 ± 6.36	0.002*	−0.82	−1.49	−0.35
Aortic pulse pressure (mmHg)	55.98 ± 7.91	52.04 ± 7.37	60.08 ± 6.28	<0.001*	−1.15	−1.74	−0.56
Augmentation index (%)	11.06 ± 6.34	9.88 ± 6.33	12.28 ± 6.25	0.180	−0.38	−0.92	0.17
AIx75 (%)	5.30 ± 7.20	4.11 ± 7.95	6.54 ± 6.26	0.232	−0.34	−0.89	0.22
Brachial-radial PWV (m/s)	8.27 ± 0.85	8.10 ± 0.80	8.45 ± 0.88	0.136	−0.42	−0.96	0.13
Carotid-femoral PWV (m/s)	6.46 ± 0.88	5.96 ± 0.64	6.98 ± 0.80	<0.001*	−1.40	−2.00	−0.79
Cardiopulmonary parameters							
HRmax (bpm)	189.10 ± 8.48	189.46 ± 8.87	188.72 ± 8.39	0.761	0.08	−0.46	0.63
HRrest (bpm)	60.24 ± 7.79	60.19 ± 8.20	60.28 ± 7.67	0.969	−0.01	−0.55	0.53
HRR (bpm)	128.86 ± 9.76	129.27 ± 10.33	128.44 ± 9.53	0.767	0.08	−0.46	0.62
$\dot{V}O_{2\max}$ (mL/kg/min)	51.77 ± 7.45	50.33 ± 8.13	53.27 ± 6.49	0.161	−0.40	−0.94	0.15
$\dot{V}O_{2}$ at VT1 (mL/kg/min)	34.45 ± 5.75	33.50 ± 5.86	35.40 ± 5.69	0.232	−0.33	−0.88	0.21
$\dot{V}O_{2}$ at VT2 (mL/kg/min)	44.30 ± 6.36	42.80 ± 6.86	45.80 ± 5.69	0.098	−0.47	−1.01	0.09
PetCO_2_ at VT1	42.9 ± 4.13	43.1 ± 4.37	42.8 ± 3.96	0.762	0.08	−0.46	0.62
PetCO_2_ at VT2	41.1 ± 4.39	41.3 ± 5	40.8 ± 3.73	0.638	0.13	−0.41	0.67

ES, effect size; 95% CI, 95% confidence interval; BMI, body mass index; PWV, pulse wave velocity; AIx75, augmentation index adjusted to 75 bpm; HRmax, maximal heart rate; HRrest, resting heart rate; HRR, heart rate reserve; $\dot{V}O_{2\max}$, maximal oxygen uptake; $\dot{V}O_{2\max}$ at VT1, oxygen uptake at first ventilatory threshold; $\dot{V}O_{2}$ at VT2, oxygen uptake at second ventilatory threshold; PetCO_2_, CO_2_ end-tidal pressure.

Data are presented as Mean ± SD. *p ≤ 0.05 between groups.

Participants were categorized into two groups based on the Seventh Report of the Joint National Committee on Prevention, Detection, and Treatment of High Blood Pressure (JNC7): an optimal blood pressure (BP) group (systolic BP < 120 mmHg and diastolic BP < 80 mmHg), and a prehypertensive group (systolic BP 120–139 mmHg and/or diastolic BP 80–89 mmHg) (28). Although more recent ACC/AHA guidelines reclassify this range as “elevated Blood pressure” or “stage 1 hypertension” (29), the term prehypertension was retained to ensure conceptual consistency with prior research examining early vascular alterations in young adults. All participants provided written informed consent before participation. The study was conducted in accordance with the principles of the Declaration of Helsinki, and all study procedures were approved by a Slovenian National Medical Ethics Committee, Ministry of Health (Ljubljana, Slovenia; reference number: 0120-63/2025-2711-3).

### 2.2 Cardiovascular assessments

#### 2.2.1 Baseline procedures and anthropometric measurements

Before arrival at the laboratory, participants received the following instructions: (a) to refrain from vigorous physical activity for 48 h prior to testing, (b) to abstain from alcohol consumption, and (c) to avoid caffeine intake on the day of testing. Female participants were tested during the follicular or luteal phase of the menstrual cycle, given the relative stability of vascular function across these phases (30). Upon arrival at the laboratory, participants were interviewed for habitual physical activity levels (GPAQ) and provided information on their previous medical and family history. Participants' body height (GPM, Siber Hegner & Co., Switzerland) and body mass (InBody 720, Biospace Co., Ltd., South Korea) were measured before vascular function assessment (31).

#### 2.2.2 Hemodynamics

Resting blood pressure was measured on the non-dominant arm after 10 min of seated rest using an automated oscillometric device (Omron M7, Japan), in accordance with current guidelines (32, 33). In accordance with the study protocol, participants attended the laboratory on three separate occasions. At each visit, blood pressure was assessed three times at 1-minute intervals, and the mean of the last two measurements was calculated. Final blood pressure values represent the average across the three visits to improve measurement reliability. All pulse wave–derived vascular measurements were performed in a quiet, temperature-controlled environment (22–25°C) following 15 min of supine rest to ensure hemodynamic stabilization.

Pulse wave analysis (PWA) was performed using an oscillometric device (Vicorder, software version 4; Skidmore Medical, United Kingdom). A brachial cuff was inflated to 65 mmHg to obtain the arterial pressure waveform via volume displacement. Central pulse pressure (AOPP) was derived using the device's generalized brachial-to-aortic transfer function. Central waveforms were calibrated using brachial systolic and diastolic blood pressure values measured immediately prior to PWA, in accordance with the manufacturer's recommendations. The device additionally enables the calculation of the augmentation index (AIx) derived from the reconstructed central pressure waveform. In addition, AIx adjusted to a standardized heart rate of 75 beats/min (AIx75) was calculated to minimize the influence of heart rate on wave reflection indices (34).

Pulse wave velocity (PWV) was assessed between the brachial and radial arteries (brPWV) and between the carotid and femoral arteries (cfPWV) using an oscillometric technique (Vicorder, Skidmore Medical, United Kingdom). For brPWV measurements, cuffs were placed at the wrist and proximally on the upper part of the right arm and were simultaneously inflated to 65 mmHg. The distance between the two cuffs was recorded for the calculation of brPWV. Pulse waveforms were captured over intervals of 3–5 s using the manufacturer's proprietary algorithm. cfPWV was measured using the same methodology, with a cuff positioned to detect the carotid pulse on the right side of the neck and a second cuff placed proximally around the right thigh. The distance between the cuffs was calculated as the distance from the suprasternal notch to the midpoint of the cuff positioned on the proximal thigh. PWV was calculated as the ratio of the direct distance between the cuffs to the time delay between the onset of the pulse wave at the proximal and distal measurement sites.

#### 2.2.3 Cardiopulmonary exercise testing

The incremental exercise test was initiated following a 5 min warm-up on a cycle ergometer (Excalibur Sport, Lode, The Netherlands) at a workload of 1 W/kg. Thereafter, a graded exercise protocol was applied, starting at 60 W, with the workload increased by 15 W every minute. Participants were instructed to maintain a cadence between 60 and 80 revolutions per minute. A subset of participants (*n* = 30; 59%) performed the exercise test on a treadmill. After a 5 min warm-up at 6 km/h, the graded exercise test commenced at 3 km/h, with the speed increased by 0.5 km/h each minute. The treadmill incline was set at 1% to better replicate the biomechanics of outdoor running. Gas exchange variables were measured throughout the test (both tests) using a metabolic analyzer/metabolic cart (COSMED Quark CPET, Italy). Heart rate was simultaneously monitored using a heart rate monitor (Polar H10, Finland). All data were recorded breath-by-breath. Participants were verbally encouraged to achieve the criteria indicative of $\dot{V}O_{2\max}$ (35).

#### 2.2.4 Ventilatory threshold analysis and intensity anchoring

Gas-exchange variables were pre-processed by excluding outliers exceeding ± 3 SD from the local mean, followed by linear interpolation of the removed values. This procedure was implemented automatically using dedicated software (Cosmed Omnia, version 2.2; Cosmed, Italy). Subsequently, breath-by-breath data were averaged into 5 s intervals for analysis. We defined VT1 according to established gas-exchange threshold criteria, whereas VT2 corresponded to the respiratory compensation point. Threshold determination was performed independently by two experienced researchers (>3 years of experience) using visual inspection and consensus in accordance with the literature (36). Briefly, VT1 was identified as the first discernible breakpoint in the $\dot{V}{\mathrm{CO}}_{2}$/$\dot{V}O_{2}$ and ${\dot{V}}_{E}$/$\dot{V}O_{2}$ relationships. Confirmation criteria included stabilization of the end-tidal PCO_2_/$\dot{V}$O_2_ and an increase in $\dot{V}$_E_/$\dot{V}$O_2_ vs. $\dot{V}$O_2_. VT2 was determined by a disproportionate rise in $\dot{V}$_E_ relative to $\dot{V}$O_2_, accompanied by a loss of stability in the $\dot{V}$_E_/$\dot{V}$CO_2_ relationship. Additionally, a decline in end-tidal PCO_2_ following a period of isocapnic buffering was used as a supportive criterion for VT2 identification. If inter-rater differences in VT1 or VT2 exceeded 10%, additional visual inspection was performed until consensus was reached. Heart rate (HR), heart rate reserve (HRR), and oxygen uptake ($\dot{V}$O_2_) values at VT1 and VT2 were directly obtained at the identified thresholds and additionally expressed relative to individual maximal values (%HRmax, %HRR, %$\dot{V}O_{2\max}$), with maximal HR and $\dot{V}$O_2_ (highest 30-s rolling average) defined as the highest values achieved during the incremental exercise test. HRR was calculated as the difference between maximal and resting HR (HRR = HRmax − HRrest), where resting HR was measured during vascular function assessment.

To derive fixed relative-intensity anchors for estimating ventilatory thresholds, mean relative values of %HRmax, %HRR, and %$\dot{V}O_{2\max}$ observed at VT1 and VT2 were calculated across the pooled sample of normotensive and prehypertensive participants and subsequently applied to individual participants' maximal HR, HRR, or $\dot{V}O_{2\max}$ values. Specifically, %HRmax and %HRR-derived anchors were used to estimate HR at VT1 and VT2, whereas %$\dot{V}O_{2\max}$-derived anchors were used to estimate $\dot{V}$O_2_ at the corresponding thresholds. Exercise intensity domains were classified according to the physiological cut-offs proposed by the American College of Sports Medicine (ACSM, 2020) and aligned with the five-level exercise intensity terminology framework (15), whereby VT1 corresponds to the transition from low- to moderate-intensity exercise and VT2 represents the transition from moderate- to high-intensity exercise. The exact cut-off values used for all relative intensity anchors are provided in Supplementary Table S1.

### 2.3 Statistical analysis

Results are presented as mean ± standard deviation (SD) when the assumption of normality was met and as median and interquartile range (IQR) when normality was violated. Differences between normotensive and prehypertensive young adults were assessed using independent-samples t-tests or the Mann–Whitney *U*-test, as appropriate. For between-group comparisons, effect sizes (ES) were quantified using Hedges’ g with corresponding 95% confidence intervals (CI). To account for potential confounding, analyses of covariance (ANCOVA) were additionally performed. For vascular outcomes, blood pressure category was included as a fixed factor, whereas sex, age, body height, body mass, and mean arterial pressure (MAP) were entered as covariates. For cardiopulmonary outcomes, blood pressure category and exercise modality were included as fixed factors, while sex was entered as a covariate. Estimated HR or $\dot{V}$O_2_ values at VT1 and VT2 were analyzed separately for each estimation method using blood pressure status–specific mean values. The Bland–Altman method was applied to determine the 95% limits of agreement (LoA) for each estimation approach. Additionally, the mean absolute error (MAE) and mean absolute percentage error (MAPE) were calculated. The distribution of participants across intensity categories was summarized using frequencies and percentages, and differences between normotensive and prehypertensive young adults were assessed using the chi-square test of independence. All statistical analyses were performed using JAMOVI (version 2.6; Jamovi, Australia) and Microsoft Excel 2021 (Microsoft Corporation, Redmond, WA, USA). Statistical significance was set at *p* < 0.05. The present study represents a secondary analysis derived from two broader projects. Therefore, the sample size was not determined *a priori* specifically for the secondary outcomes examined in the current manuscript.

## 3 Results

Demographic, anthropometric, clinical, and cardiopulmonary characteristics of normotensive and prehypertensive young adults are summarized in Table 1. After accounting for comparable levels of habitual physical activity, as additionally confirmed by the GPAQ questionnaire (*p* = 0.434), no meaningful differences were observed between groups in cardiopulmonary variables. In contrast, between-group differences were primarily evident in clinical measures related to CV risk, with prehypertensive individuals exhibiting significantly higher systolic blood pressure (*p* < 0.001), AOPP (*p* < 0.001), and cfPWV (*p* < 0.001). No significant between-group differences were observed for diastolic blood pressure (*p* = 0.329), AIx (*p* = 0.180), AIx75 (*p* = 0.232), or brPWV (*p* = 0.136). Differences were also observed in anthropometric characteristics (body height and body mass), reflecting the lower proportion of females in the prehypertensive group. Given this imbalance in sex distribution, additional ANCOVA analyses were performed (Supplementary Table S3), with blood pressure category specified as the primary fixed factor, while sex, age, body mass, height, and mean arterial pressure (MAP) were entered as covariates. Furthermore, after adjustment for covariates, blood pressure category remained significantly associated with cfPWV (*p* = 0.001) and AOPP (*p* = 0.045).

Mean exercise intensity anchors at VT1 and VT2 are presented in Table 2. Relative heart rate (bpm), %HRmax, %HRR, and %$\dot{V}O_{2\max}$ at VT1 and VT2 did not differ significantly between normotensive and prehypertensive young adults (*p* = 0.197–0.716). Similarly, these relative intensity markers did not differ when analyses were stratified by sex (Supplementary Table S2). In contrast, females demonstrated a significantly lower resting heart rate (*p* = 0.011), lower absolute $\dot{V}O_{2\max}$, lower absolute oxygen uptake at both ventilatory thresholds (all *p* < 0.001), and lower end-tidal carbon dioxide pressure (PetCO₂) at VT1 (*p* = 0.003), consistent with established sex-related physiological differences. These findings were further supported by ANCOVA analyses of cardiopulmonary variables (Supplementary Tables S4, S5), which revealed no significant main effects of blood pressure category after adjustment for sex, while accounting for exercise modality during CPET.

**Table 2:** Mean values at the first and second ventilatory thresholds for normotensive and prehypertensive young adults.

	All (*N* = 51)	Normotension (*N* = 26)	Prehypertension (*N* = 25)	*p*—value
VT1				
HR (bpm)	149.89 ± 13.31	152.08 ± 14.21	147.60 ± 12.46	0.238
HR (%/max)	79.24 ± 7.78	80.20 ± 5.66	78.23 ± 5.94	0.232
HRR (%/max)	71.5 (15.3)	73.3 (15)	68.9 (14.1)	0.197
$\dot{V}$O_2_ (%/max)	66.3 (4.83)	66.6 (4.01)	65.8 (5.42)	0.543
VT2				
HR (bpm)	176 (11.5)	176 (12)	175 (11)	0.509
HR (%/max)	92.5 (2.70)	93 (2.61)	91.8 (2.23)	0.386
HRR (%/max)	89.2 (4.65)	89 (3.61)	88.1 (5.02)	0.434
$\dot{V}$O_2_ (%/max)	86 (4.02)	85.9 (4.2)	86 (3.68)	0.716

Values are presented as Mean ± SD when the assumption of normality was met, and as Median (IQR) when the normality assumption was violated.

HR, heart rate; HRR, heart rate reserve; $\dot{V}$O_2_, oxygen uptake.

Exercise intensity classification at VT1 and VT2 is presented in Table 3, with the relative intensity anchors used for categorization summarized in Supplementary Table S1. Regardless of the intensity marker applied (HR-derived %HRmax and %HRR anchors or the $\dot{V}$O_2_-derived %$\dot{V}O_{2\max}$ anchor), no statistically significant differences were observed between normotensive and prehypertensive individuals in the distribution across intensity zones (all *p* > 0.05). Exercise intensity at VT1 was predominantly classified as high intensity, which is inconsistent with guideline-based expectations that VT1 represents the transition from low- to moderate-intensity exercise, indicating a pronounced overestimation of relative intensity at this threshold. In contrast, intensity classification at VT2 was more consistent with theoretical expectations, as VT2 is assumed to represent the transition from moderate- to high-intensity exercise, and accordingly most participants were classified within the high-intensity zone, with only a small proportion categorized as very high intensity.

**Table 3:** First and second ventilatory thresholds and corresponding cardiopulmonary exercise testing parameters in normotensive and prehypertensive young adults.

	Normotensive (*N* = 26)	Prehypertensive (*N* = 25)	Normotensive (*N* = 26)	Prehypertensive (*N* = 25)
Correspondence of exercise intensity according to ACSM (2020) and the aligned five-level exercise intensity terminology				
	VT1_%HRmax		VT2_%HRmax	
Very low	–	–	–	–
Low	–	–	–	–
Moderate	19.2%	40%	–	–
High	80.8%	60%	88.5%	92%
Very high	–		11.5%	8%
	VT1_%HRR		VT2_%HRR	
Very low	–	–	–	–
Low	–	–	–	–
Moderate	7.7%	16%	–	–
High	92.3%	84%	53.8%	64%
Very high	–	–	46.2%	36%
	VT1_%$\dot{V}O_{2\max}$		VT2_%$\dot{V}O_{2\max}$	
Very low	–	–	–	–
Low	–	–	–	–
Moderate	15.4%	24%	–	–
High	84.6%	76%	96.2%	100%
Very high	–	–	3.8%	0%

Data are expressed as percentages of subjects stratified according to exercise intensity for each cardiopulmonary exercise testing parameter, using ACSM (2020) physiological cut-offs and the five-level terminology proposed by Bishop et al. (2025).

HR, heart rate; HRR, heart rate reserve; $\dot{V}$O_2_, oxygen uptake.

Agreement and accuracy metrics for estimated ventilatory thresholds derived from population-based anchors and guideline-based anchors are summarized in Table 4. As no significant between-group differences were identified, data are presented as pooled values. Among the population-derived anchors, %$\dot{V}O_{2\max}$ demonstrated the smallest error magnitude and narrowest limits of agreement at VT1, whereas %HRmax showed the best overall agreement at VT2. In contrast, ACSM guideline-based anchors consistently exhibited larger errors and wider limits of agreement across both thresholds. Overall, estimation accuracy was greater at VT2 than at VT1, as reflected by lower MAE and MAPE values and narrower limits of agreement across all evaluated anchors.

**Table 4:** Mean bias, 95% limits of agreement (LoA), mean absolute error (MAE), and mean absolute percentage error (MAPE) for population-derived and ACSM (2020) guideline-based exercise-intensity anchors (%HRmax, %HRR, and %$\dot{V}O_{2\max}$) at the first and second ventilatory thresholds in pooled data from normotensive and prehypertensive individuals.

Anchor type	Threshold	Estimation method	Bias	95% LoA	MAE	MAPE
Estimation based on population-derived anchors						
HR-derived anchors	VT1	HR _est_%HRmax	−0.04 ± 10.95 bpm	−21.42 to 21.50 bpm	9.27 ± 5.68 bpm	6.34 ± 4.26%
		HR_est_%HRR	−0.15 ± 11.04 bpm	−21.48 to 21.79 bpm	9.51 ± 5.43 bpm	6.49 ± 4.09%
	VT2	HR _est_%HRmax	−0.03 ± 5.19 bpm	−10.15 to 10.21 bpm	3.92 ± 3.36 bpm	2.30 ± 2.13%
		HR_est_%HRR	−0.04 ± 5.17 bpm	−10.09 to 10.18 bpm	4.00 ± 3.21 bpm	2.36 ± 2.04%
$\dot{V}$O_2_-derived anchor	VT1	$\dot{V}$O_2__est_%$\dot{V}O_{2\max}$	0.24 ± 3.00 mL/kg/min	−5.64 to 6.11 mL/kg/min	2.11 ± 2.12 mL/kg/min	6.30 ± 6.96%
	VT2	$\dot{V}$O_2__est_%$\dot{V}O_{2\max}$	0.21 ± 1.95 mL/kg/min	−3.61to 4.03 mL/kg/min	1.40 ± 1.36 mL/kg/min	3.17 ± 3.26%
Estimation based on ACSM (2020) guideline anchors						
HR-derived anchors	VT1	HR _est_%HRmax	−28.86 ± 11.16 bpm	−7.00 to −50.72 bpm	28.86 ± 11.16 bpm	18.78 ± 6.19%
		HR_est_%HRR	−38.10 ± 11.89 bpm	−14.80 to −61.40 bpm	38.10 ± 11.89 bpm	24.99 ± 6.28%
	VT2	HR _est_%HRmax	−28.43 ± 5.49 bpm	−17.70 to −39.20 bpm	28.43 ± 5.49 bpm	16.24 ± 2.64%
		HR_est_%HRR	−36.48 ± 6.17 bpm	−24.40 to −48.60 bpm	36.48 ± 6.17 bpm	20.87 ± 2.87%
$\dot{V}$O_2_-derived anchor	VT1	$\dot{V}O_{2\max}$O_2__est_%$\dot{V}O_{2\max}$	−10.64 ± 3.37 mL/kg/min	−17.24 to −4.03 mL/kg/min	10.6 ± 3.37 mL/kg/min	30.3 ± 6.39%
	VT2	$\dot{V}O_{2\max}$O_2__est_%$\dot{V}O_{2\max}$	−11.18 ± 2.36 mL/kg/min	−15.81 to −6.54 mL/kg/min	11.2 ± 2.36 mL/kg/min	25.10 ± 3.37%

HR_est, estimated heart rate; HRmax, maximal heart rate; HRR, heart rate reserve; $\dot{V}$O_2_, oxygen uptake; $\dot{V}O_{2\max}$, maximal oxygen uptake; VT1, first ventilatory threshold; VT2, second ventilatory threshold; HR_est_%HRmax, heart rate estimated from %HRmax; HR_est_%HRR, heart rate estimated from %HRR; $\dot{V}$O_2__est_%$\dot{V}O_{2\max}$, oxygen uptake estimated from %$\dot{V}O_{2\max}$. HR-derived anchors were evaluated against measured HR at VT1 and VT2, whereas $\dot{V}$O_2_-derived anchors were evaluated directly against measured $\dot{V}$O_2_ at the corresponding thresholds.

## 4 Discussion

The primary objective of this study was to compare vascular and hemodynamic characteristics, $\dot{V}O_{2\max}$, and ventilatory thresholds between normotensive and prehypertensive young, physically active adults. A secondary objective was to assess the accuracy of commonly used fixed relative intensity anchors for estimating ventilatory thresholds and to determine their alignment with guideline-based intensity classifications. The principal findings indicate that prehypertension is associated with early central vascular alterations despite preserved $\dot{V}O_{2\max}$ and unaltered ventilatory threshold–derived indices of exercise performance. Moreover, fixed relative intensity anchors demonstrated limited accuracy in approximating ventilatory thresholds, independent of blood pressure status.

### 4.1 Subclinical vascular alterations

Although brachial blood pressure remains the clinical reference standard for the diagnosis of hypertension, important information regarding vascular health may not be fully captured by peripheral blood pressure alone, particularly in relation to arterial stiffness (37). In this context, prehypertension represents an intermediate blood pressure category between normotension and overt hypertension, in which subclinical vascular alterations may already be present (38). In the present study, prehypertension was associated with less favourable central vascular characteristics compared with normotension. Despite comparable age, BMI, $\dot{V}O_{2\max}$, and habitual physical activity levels, individuals with prehypertension exhibited higher AOPP and cfPWV, indicating greater central arterial stiffness. These findings are consistent with previous reports demonstrating associations between elevated blood pressure within the prehypertensive range and less favourable central vascular characteristics (38–41), while emphasizing central (aortic) rather than peripheral indices of vascular function.

The observed pattern, in which cfPWV and AOPP differed between groups, while peripheral indices (AIx, AIx75, and brPWV) did not, is consistent with the notion that central and peripheral arterial beds respond differently to blood pressure-related stimuli. Central arterial stiffness is generally considered less responsive than peripheral arterial properties, reflecting inherent differences in the structural and temporal characteristics of central and peripheral vascular beds (42, 43). Specifically, central arterial stiffness is predominantly driven by structural remodelling of the arterial wall, including elastin degradation and collagen accumulation, processes that are considered less readily reversible through habitual physical activity than peripheral vascular adaptations (44–46). In contrast, peripheral arterial properties exhibit greater intrinsic plasticity and may therefore be more receptive to exercise-induced modification. In line with this interpretation, exercise training in young prehypertensive individuals has previously been shown to improve peripheral arterial stiffness indices, including AIx and AIx75, without necessarily altering central cfPWV values (47).

Collectively, these findings provide evidence of early central vascular alterations associated with prehypertension in young, physically active adults. Notably, these differences were observed despite comparable levels of cardiopulmonary fitness, underscoring a potential dissociation between vascular health and aerobic capacity in this population.

### 4.2 Aerobic capacity and exercise performance

Despite the presence of less favourable central vascular characteristics, prehypertensive individuals did not differ from their normotensive counterparts in $\dot{V}O_{2\max}$ or ventilatory threshold–derived indices of exercise performance (*p* > 0.05). Both $\dot{V}O_{2\max}$ and ventilatory thresholds were comparable between groups, indicating that young, physically active adults with prehypertension maintain intact central and peripheral components of oxygen delivery and utilisation. In addition, no group differences were observed in resting heart rate, heart rate responses at VT1, VT2, or at maximal exertion, nor in heart rate reserve, suggesting preserved autonomic and chronotropic regulation during exercise in this population. Collectively, these findings suggest that early vascular alterations associated with elevated blood pressure do not necessarily translate into detectable impairments in maximal or submaximal exercise performance indices at this stage.

From a physiological perspective, this dissociation between vascular health and $\dot{V}O_{2\max}$ is not unexpected. Previous work by Jung and colleagues (10) reported no association between exercise capacity and blood pressure when comparing normotensive and prehypertensive adults, whereas attenuated exercise capacity has primarily been observed in individuals with established hypertension compared with normotensive controls (48). Although the mechanisms underlying these differences are not fully elucidated, elevated heart rate secondary to autonomic disturbance has been proposed as a potential contributing factor in hypertension (10). In contrast, the absence of differences in heart rate responses across all exercise stages in the present study suggests that such autonomic alterations are not yet evident in young, physically active individuals with prehypertension. This supports the interpretation that aerobic capacity in this population is predominantly constrained by cardiac output (10), skeletal muscle oxidative capacity (49, 50), and neuromuscular factors, while moderate increases in central arterial stiffness were not accompanied by detectable reductions in exercise performance in the present cohort (51, 52).

Furthermore, aerobic capacity is known to decline with advancing age, and this decline is associated with increased risks of hypertension and all-cause mortality (52–55). The relatively young age and high training status of the present cohort likely explain the preserved exercise performance and heart rate regulation observed in prehypertensive individuals. Habitual physical activity and training-related adaptations may mitigate the functional consequences of early vascular dysfunction by maintaining favourable endothelial function, capillary density, and mitochondrial efficiency (56, 57). Consequently, cardiopulmonary fitness alone may be insufficiently sensitive to detect early cardiovascular risk in physically active populations (58). From a clinical and preventive perspective, the present findings indicate that reliance on brachial blood pressure and cardiopulmonary fitness alone may underestimate early cardiovascular risk in physically active young adults. The inclusion of central vascular assessments, such as cfPWV, may therefore provide complementary information for the early identification of subclinical vascular ageing in this population.

### 4.3 Exercise intensity domains and ventilatory thresholds

The patterns observed in the correspondence between ventilatory thresholds and fixed relative intensity anchors demonstrate a clear differential alignment between VT1 and VT2, which can be explained by both the physiological characteristics of the thresholds and the limitations inherent to fixed relative intensity anchors. Notably, the most pronounced misclassification in the present study was observed at VT1. Although VT1 is generally expected to correspond to the transition between low- and moderate-intensity domains (15, 59), it was systematically classified within the high-intensity domain across all fixed relative intensity anchors. When expressed relative to fixed intensity anchors, VT1 was predominantly classified within the high-intensity domain, indicating a systematic upward shift in intensity categorization at this threshold. In contrast, VT2 demonstrated substantially better agreement with guideline-defined intensity domains (15, 59), highlighting a threshold-specific difference in the validity of commonly used relative intensity anchors. These findings are consistent with previous reports demonstrating that fixed relative intensity anchors systematically overestimate the effective exercise intensity when ventilatory thresholds are used as reference points (11, 20, 60, 61). In the present study, this overestimation was more pronounced at VT1, whereas VT2 showed a comparatively closer correspondence with guideline-defined domains. This pattern is in line with contemporary conceptual frameworks of exercise intensity domains, which emphasize that VT1 demarcates the transition from light- to moderate-exercise, whereas VT2 represents the boundary between moderate- and high-intensity exercise (19, 20). The broader interindividual dispersion of VT1 when expressed relative to %$\dot{V}O_{2\max}$, %HRmax, or %HRR limits the precision of fixed relative intensity anchors in capturing this transition at the individual level (22). In contrast, VT2 typically occurs within a narrower relative intensity range (16), resulting in more consistent alignment with guideline-defined intensity domains. Importantly, given the comparable training status of normotensive and prehypertensive participants, the absence of between-group differences in domain distribution suggests that the observed misclassification reflects methodological constraints of fixed relative anchors rather than blood pressure status *per se*.

The extent of this misclassification is further characterised by the agreement analyses presented in Table 4. Given that neither blood pressure status nor sex materially influenced the relative positioning of VT1 and VT2 when expressed as %HRmax, %HRR, or %$\dot{V}O_{2\max}$ in our cohort (Supplementary Table S2), agreement analyses were therefore performed on pooled data, consistent with recent approaches used to benchmark anchor-derived estimates against criterion thresholds (14). Notably, males exhibited higher absolute $\dot{V}O_{2\max}$ and oxygen uptake at both VT1 and VT2, as consistently reported in the existing literature (62–64). These differences did not translate into systematic sex-related shifts in the relative positioning of ventilatory thresholds when expressed relative to fixed intensity anchors, thereby supporting the validity of pooled agreement analyses and indicating that anchor-related misclassification is unlikely to be explained by sex-related differences.

Across all fixed relative intensity anchors, agreement metrics indicated a clear threshold-dependent pattern, with consistently larger bias and error estimates observed at VT1 compared with VT2. Specifically, MAE and MAPE were markedly higher for VT1 across %HRmax, %HRR, and %$\dot{V}O_{2\max}$, accompanied by wider limits of agreement, reflecting substantial interindividual variability in anchor-based estimation of the low-to-moderate intensity transition. In contrast, agreement at VT2 was characterised by smaller bias, narrower LoA, and lower MAE and MAPE values, indicating a more stable and predictable relationship between fixed relative intensity anchors and the VT2. Notably, guideline-based anchors derived from the ACSM recommendations (59) exhibited greater systematic bias than cohort-derived mean anchors, particularly at VT1, whereas differences between anchor types were attenuated at VT2. Collectively, the agreement analyses indicate that, although estimation error differs between VT1 and VT2, guideline-based anchors exhibit substantially greater bias than population-derived mean anchors, particularly at lower exercise intensity domains. Importantly, when maximal effort is achieved, and $\dot{V}O_{2\max}$ is directly measured, expressing intensity relative to %$\dot{V}O_{2\max}$ demonstrated the lowest estimation error at VT1, whereas %HRmax-based anchors showed the best overall agreement at VT2. This approach is primarily applicable in laboratory settings, whereas in non-laboratory contexts, %HRmax-based anchors may represent the most feasible and practically applicable alternative despite their slightly lower precision. Importantly, these findings do not undermine the value of heart rate as a continuous indicator of internal load, but rather highlight that fixed-percentage HR-anchors are insufficient substitutes for individually determined physiological thresholds. Given the central role of accurately defining exercise intensity domains and the threshold-dependent limitations of fixed relative intensity anchors demonstrated in the present study, it is essential to identify feasible and valid approaches for estimating exercise intensity with sufficient accuracy. In this context, increasing emphasis should be placed on the development and application of wearable, non-invasive technologies capable of capturing individual, intensity-dependent responses to exercise intensity under real-world conditions. Such approaches offer the potential to characterise exercise intensity based on objective, individualised signals, rather than relying on fixed relative intensity anchors (14, 15, 21) or subjective estimations (65). These approaches include the use of non-invasive markers, such as thresholds derived from heart rate variability (66, 67), as well as wearable technologies such as near-infrared spectroscopy (NIRS), which has demonstrated promising potential for the determination of ventilatory thresholds (24). Accordingly, future exercise-intensity guidelines may benefit from greater consideration of individual variability in training and health status, with particular attention to methods that allow more individualized delineation of intensity domains. Threshold-based approaches represent one such strategy, as they may help account for interindividual differences in internal load responses and thereby facilitate more individualized and informed exercise prescription (13).

### 4.4 Clinical and research implications

From a clinical perspective, in physically active young adults, prehypertension may coexist with early central vascular alterations despite preserved aerobic capacity. These findings suggest that not only engaging in exercise but also how exercise intensity is prescribed may be important, as commonly used fixed relative intensity anchors (e.g., %HRmax, %HRR, %$\dot{V}O_{2\max}$) may not accurately capture physiological exercise-intensity domains. When feasible, exercise prescription guided by ventilatory thresholds may provide a more physiologically appropriate approach for individualized exercise guidance.

### 4.5 Limitations

This study also has several limitations. First, the cross-sectional design precludes causal inference regarding the directionality between habitual physical activity status and early vascular alterations in this cohort. However, the primary aim was to characterise associations rather than causal mechanisms, and the observed patterns provide a rationale for future longitudinal investigations. Second, the present study represents a secondary exploratory analysis and was therefore not specifically powered *a priori* for all outcomes examined in the current manuscript. Although the sample size was modest and the sex distribution differed between groups, sex was included as a covariate in all adjusted analyses to reduce the potential influence of unequal group distribution. Third, central hemodynamic parameters were derived using an oscillometric transfer-function approach rather than applanation tonometry, which is often considered the reference method. Although this technique is widely used and validated, it may yield differences in absolute central pressure and cfPWV estimates compared with tonometry-based methods and should therefore be considered when interpreting the magnitude of aortic pulse pressure values and absolute cfPWV values. Finally, CPET was performed using two exercise modalities (cycle ergometer and treadmill), both of which were retained in the current analyses. Exercise modality was selected according to participants' primary training background and familiarity with the testing modality, whereby recreational runners predominantly performed treadmill CPET and recreational cyclists performed cycle ergometer CPET, in order to facilitate maximal exercise performance and reduce modality-specific limitations associated with unfamiliar movement patterns related to exercise economy, neuromuscular coordination, and local muscular fatigue in non-specific exercise modalities. To account for potential modality-related effects, exercise modality was included as an adjustment factor in the statistical analyses (68, 69). Nevertheless, physiological differences between treadmill- and cycle ergometer–based CPET assessments are well documented, particularly concerning peak $\dot{V}$O_2_ and threshold-related responses (69), and may therefore have contributed to variability in maximal and threshold-derived cardiopulmonary responses. At the same time, this approach reflects real-world testing conditions and supports the applicability of the present findings across commonly used exercise testing modalities, while precluding the use of a single external load–based fixed anchor (e.g., speed or power output) for threshold estimation across participants.

## 5 Conclusion

In young, physically active adults, prehypertension is associated with early central vascular alterations, reflected by increased central arterial stiffness, despite preserved aerobic capacity and normal ventilatory thresholds. These findings indicate that cardiopulmonary fitness remains preserved at this stage, suggesting that early vascular alterations may precede detectable impairments in aerobic capacity. In addition, commonly used fixed relative intensity anchors (e.g., %HRmax, %HRR, %$\dot{V}O_{2\max}$) demonstrated limited validity for estimating exercise-intensity domains corresponding to ventilatory thresholds, with substantial and threshold-dependent misclassification that was most pronounced at VT1 and less evident at VT2. Among the evaluated approaches, %$\dot{V}O_{2\max}$ demonstrated the lowest estimation error at VT1, whereas %HRmax showed the best overall agreement at VT2, although all fixed relative intensity anchors remained inferior to exercise prescription based on ventilatory thresholds. Collectively, these findings highlight the importance of assessing central arterial stiffness for the early detection of vascular alterations associated with prehypertension and support more physiologically grounded approaches for exercise-intensity prescription when direct threshold assessment is not available.

## Data Availability

The raw data supporting the conclusions of this article will be made available by the authors, without undue reservation.
