# Supplementary material for: Prehypertension in young physically active adults: early vascular alterations and implications for exercise-intensity prescription
Source: Front Cardiovasc Med. 2026 Jun 29;13:1853384. doi: 10.3389/fcvm.2026.1853384 (PMC13357653; doi:10.3389/fcvm.2026.1853384)
Supplement: Supplementary file 1 [file Datasheet1.docx]

Contents

[*S*1 *–* Methodology used for categorization of physical exercise interventions 2](#_Toc230085228)

[S2 *–* Additional statistical analyses 3](#_Toc230085229)

[*S3* – References 7](#_Toc230085230)

# *S*1 *–* Methodology used for categorization of physical exercise interventions

Supplementary Table 1 represents relative intensity anchors expressed as %HRmax, %HRR, and %V̇O_2max_, aligned with ACSM-based cut-offs and the five-level exercise intensity classification framework, with VT1 defined as the transition between low- and moderate-intensity and VT2 as the transition between moderate- and high-intensity.

Supplementary Table 1. Exercise intensity categories derived from maximal exercise testing and ventilatory thresholds according to ACSM (2020) and Bishop et al. (2025)

| **Intensity category**  Bishop et al. (2025) |  | **%HRmax**  **ACSM (2020)** | **%HRR**  **ACSM (2020)** | **%V̇O_2max_**  **ACSM (2020)** |
| --- | --- | --- | --- | --- |
| Very low |  | <57 | <30 | <37 |
| Low |  | 57–63 | 30–39 | 37–45 |
|  | **VT1** | 64 | 40 | 46 |
| Moderate |  | 64–76 | 40–59 | 46–63 |
|  | **VT2** | 77 | 60 | 64 |
| High |  | 77–95 | 60–89 | 64–90 |
| Very high |  | >86 | ≥90 | ≥91 |

*Abbreviations:* HRmax, maximal heart rate; HRR, heart rate reserve; VO₂max, maximal oxygen uptake; VT1, first ventilatory threshold; VT2, second ventilatory threshold.

# S2 *–* Additional statistical analyses

Supplementary Table 2 presents sex-specific descriptive statistics for cardiopulmonary variables at the first (VT1) and second (VT2) ventilatory thresholds. Between-group differences were evaluated using independent-samples t-tests when the assumption of normality was met, and Mann–Whitney U tests when normality was violated.

Supplementary Table 2. Mean values (±SD) at the first (VT1) and second (VT2) ventilatory thresholds for men and women

|  | | | All (51) | | Men (N=32) | | Woman (N=19) | | p - value |
| --- | --- | --- | --- | --- | --- | --- | --- | --- | --- |
| **Cardiopulmonary parameters** | | | | | | | | | |
| HRmax (bpm) | 189.10 ± 8.48 | | | 188.8 ± 8.29 | | 189.6 ± 9.22 | | 0.761 | |
| HRrest (bpm) | 60.24 ± 7.79 | | | 58.1 ± 6.32 | | 63.8 ± 9.03 | | 0.011* | |
| HRR (bpm) | 128.86 ± 9.76 | | | 130.7 ± 9.69 | | 125.8 ± 9.61 | | 0.086 | |
| V̇O_2max_ (ml/kg/min) | 51.77 ± 7.45 | | | 55.6 ± 6.12 | | 45.4 ± 4.54 | | < 0.001* | |
| VO_2_ at VT1 (ml/kg/min) | 34.45 ± 5.75 | | | 36.8 ± 5.49 | | 30.4 ± 3.82 | | < 0.001* | |
| VO_2_ at VT2 (ml/kg/min) | 44.30 ± 6.36 | | | 47.3 ± 5.60 | | 39.3 ± 4.29 | | < 0.001* | |
| PetCO2 at VT1 | 42.9 ± 4.13 | | | 44.2 ± 4.14 | | 40.8 ± 3.19 | | 0.003* | |
| PetCO2 at VT2 | 41.1 ± 4.39 | | | 41.8 ± 4.45 | | 39.8 ± 4.11 | | 0.128 | |
| **VT1** | | | | | | | | | |
| HR (bpm) | | 149.89 ± 13.31 | | | 148.7 ± 13.07 | | 151.9 ± 14.17 | | 0.415 |
| HR (%/max) | | | 79.24 ± 7.78 | | 78.7 ± 5.92 | | 80.1 ± 5.72 | | 0.441 |
| HRR (%/max) | | | 69.45 ± 8.55 | | 71.5 (15.1) | | 71.4 (14.7) | | 0.704 |
| VO₂ (%/max) | | | 66.51 ± 5.72 | | 66 (6.61) | | 66.6 (3.63) | | 0.493 |
| **VT2** | | | | | | | | | |
| HR (bpm) | | | 174.04 ± 9.83 | | 175 (11.5) | | 176 (11) | | 0.250 |
| HR (%/max) | | | 92.02 ± 2.78 | | 91.9 (3.1) | | 92.9 (2.04) | | 0.235 |
| HRR (%/max) | | | 88.28 ± 4.04 | | 88.3 (5.24) | | 89.3 (3.14) | | 0.565 |
| V̇O₂ (%/max) | | | 85.97 ± 5.31 | | 85.6 (4.53) | | 87.2 (3.57) | | 0.249 |

*Note:* Values are presented as Mean ± SD when the assumption of normality was met, and as Median (IQR) when the normality assumption was violated; *p ≤ 0.05 between groups.

Abbreviations: BMI, body mass index; PWV, pulse wave velocity; HRmax, maximal heart rate; HRrest, resting heart rate; HRR, heart rate reserve; VO2max, maximal oxygen uptake; VO2 at VT1, oxygen uptake at first ventilatory threshold; VO2 at VT2, oxygen uptake at second ventilatory threshold; PetCO2, CO2 end-tidal pressure

Analyses presented in Supplementary Table 3 were conducted using analysis of covariance (ANCOVA), with blood pressure category specified as the primary fixed factor, while sex, age, height, body mass, and mean arterial pressure (MAP) were entered as covariates. Analyses presented in Supplementary Tables 4 and 5 were also performed using analysis of covariance (ANCOVA), with blood pressure category and exercise modality used during the CPET test included as fixed factors, whereas sex was entered as a covariate

***Supplementary Table 3.*** ***Multivariable ANCOVA results for vascular measures***

| **Predictor** | **cfPWV** | **baPWV** | **AOPP** | **AIx** | **AIx75** |
| --- | --- | --- | --- | --- | --- |
| BP category | 11.55; 0.001*; 0.166 | 0.00; 0.988; 0.000 | 4.27; 0.045*; 0.083 | 0.25; 0.622; 0.004 | 1.07; 0.307; 0.017 |
| Sex | 3.17; 0.082; 0.046 | 0.09; 0.766; 0.001 | 1.23; 0.273; 0.024 | 4.64; 0.037*; 0.079 | 9.12; 0.004*; 0.145 |
| Age | 6.31; 0.016*; 0.091 | 7.13; 0.011*; 0.095 | 0.69; 0.412; 0.013 | 0.19; 0.661; 0.003 | 0.44; 0.511; 0.007 |
| Weight | 3.61; 0.064; 0.052 | 5.15; 0.028*; 0.068 | 1.03; 0.315; 0.020 | 7.63; 0.008*; 0.130 | 7.27; 0.010*; 0.115 |
| Height | 0.38; 0.539; 0.006 | 1.19; 0.281; 0.016 | 0.00; 0.983; 0.000 | 0.12; 0.727; 0.002 | 0.08; 0.774; 0.001 |
| MAP | 0.54; 0.468; 0.008 | 17.67; <0.001*; 0.235 | 0.28; 0.601; 0.005 | 1.86; 0.180; 0.032 | 1.10; 0.300; 0.017 |

*Abbreviations:* cfPWV, carotid–femoral pulse wave velocity; brPWV, brachial–radial pulse wave velocity; AOPP, aortic pulse pressure; AIx, augmentation index; AIx75, augmentation index adjusted to a heart rate of 75 bpm; BP, blood pressure; MAP, mean arterial pressure.

*Note:* Values are reported as F-statistics; p-values; partial eta squared (η^2^). Statistical significance was set at p < 0.05 and is indicated by *.

Supplementary Table 4. Multivariable ANCOVA results for cardiopulmonary measures

| **Predictor** | **HRmax** | **HRmin** | **HRR** | **V̇O₂max** | **V̇O₂ at VT1** | **V̇O₂ at VT2** | **PetCO₂ at VT1** | **PetCO₂ at VT2** |
| --- | --- | --- | --- | --- | --- | --- | --- | --- |
| BP category | 0.18; 0.671; 0.004 | 1.58; 0.214; 0.033 | 1.91; 0.173; 0.039 | 0.91; 0.344; 0.019 | 0.51; 0.478; 0.011 | 0.11; 0.742; 0.002 | 3.29; 0.076; 0.065 | 1.46; 0.232; 0.030 |
| Exercise modality | 10.27; 0.002*; 0.179 | 0.40; 0.533; 0.008 | 5.14; 0.028*; 0.099 | 0.31; 0.583; 0.006 | 4.05; 0.050*; 0.079 | 1.02; 0.318; 0.021 | 2.59; 0.114; 0.052 | 1.10; 0.299; 0.023 |
| Sex | 0.16; 0.694; 0.003 | 8.82; 0.005*; 0.158 | 4.19; 0.046*; 0.082 | 35.89; <0.001*; 0.433 | 18.08; <0.001*; 0.278 | 23.31; <0.001*; 0.332 | 14.73; <0.001*; 0.239 | 4.07; 0.049*; 0.080 |

*Abbreviations:* HRmax, maximal heart rate; HRmin, resting (minimal) heart rate; HRR, heart rate reserve; VO₂max, maximal oxygen uptake; VO₂ at VT1, oxygen uptake at the first ventilatory threshold; VO₂ at VT2, oxygen uptake at the second ventilatory threshold; PetCO2, CO_2_ end-tidal pressure.

*Note:* Values are reported as F-statistics; p-values; partial eta squared (η^2^). Statistical significance was set at p < 0.05 and is indicated by *.

Supplementary Table 5. Results of ANCOVA on cardiopulmonary variables at VT1 and VT2

| **Predictor** | **HR (bpm)** | **HR (%/max)** | **HRR (%/max)** | **V̇O₂ (%/max)** |  | **HR (bpm)** | | **HR (%/max)** | **HRR (%/max)** | **V̇O₂ (%/max)** |
| --- | --- | --- | --- | --- | --- | --- | --- | --- | --- | --- |
|  | **VT1** | | | |  | | **VT2** | | | |
| BP category | 1.64; 0.206; 0.034 | 1.34; 0.252; 0.028 | 1.89; 0.176; 0.039 | 0.01; 0.910; 0.000 |  | 0.16; 0.693; 0.003 | | 0.01; 0.942; 0.000 | 0.08; 0.784; 0.002 | 2.20; 0.144; 0.045 |
| Sex | 0.36; 0.552; 0.008 | 0.19; 0.664; 0.004 | 0.02; 0.890; 0.000 | 0.37; 0.545; 0.008 |  | 1.04; 0.312; 0.022 | | 1.34; 0.253; 0.028 | 0.54; 0.468; 0.011 | 4.20; 0.046*; 0.082 |
| Exercise modality | 13.51; <0.001*; 0.223 | 5.49; 0.023*; 0.105 | 6.10; 0.017*; 0.115 | 7.69; 0.008*; 0.141 |  | 14.47; <0.001*; 0.235 | | 4.06; 0.050*; 0.080 | 4.53; 0.039*; 0.088 | 1.60; 0.213; 0.033 |

*Abbreviations:* HR, heart rate; HRmax, maximal heart rate; HRR, heart rate reserve; VO₂, oxygen uptake; VT1, first ventilatory threshold; VT2, second ventilatory threshold; BP, blood pressure.

*Note:* Values are reported as F-statistics; p-values; partial eta squared (η^2^). Statistical significance was set at p < 0.05 and is indicated by *.

# *S3* – References

1. BISHOP DJ, BECK B, BIDDLE SJH, et al. Physical Activity and Exercise Intensity Terminology: A Joint American College of Sports Medicine (ACSM) Expert Statement and Exercise and Sport Science Australia (ESSA) Consensus Statement. *Med Sci Sports Exerc*. 2025;57(11):2599-2613. doi:10.1249/MSS.0000000000003795
2. Liguori, G. (2022). *ACSM’s Guidelines for Exercise Testign and Prescription*. https://books.google.com/books/about/ACSM_s_Guidelines_for_Exercise_Testing_a.html?hl=sl&id=hTKvzgEACAAJ
